# Doctors and Vampires in Sub-Saharan Africa: Ethical Challenges in Clinical Trial Research

**DOI:** 10.4269/ajtmh.13-0630

**Published:** 2014-08-06

**Authors:** Koen Peeters Grietens, Joan Muela Ribera, Annette Erhart, Sarah Hoibak, Raffaella M. Ravinetto, Charlotte Gryseels, Susan Dierickx, Sarah O'Neill, Susanna Hausmann Muela, Umberto D'Alessandro

**Affiliations:** Department of Public Health, Institute of Tropical Medicine, Antwerp, Belgium; School of International Health Development, Nagasaki University; Partners for Applied Social Sciences (PASS) International, Tessenderlo, Belgium; Department of Biomedical Sciences, Institute of Tropical Medicine, Antwerp, Belgium; Global Fund to Fight AIDS, Tuberculosis and Malaria, Geneva, Switzerland; Department of Clinical Sciences, Institute of Tropical Medicine, Antwerp, Belgium; Department of Pharmaceutical and Pharmacological Sciences, KU Leuven, Belgium; Medical Research Council, Fajara, The Gambia; London School of Tropical Medicine and Hygiene, London, United Kingdom

## Abstract

Collecting blood samples from individuals recruited into clinical research projects in sub-Saharan Africa can be challenging. Strikingly, one of the reasons for participant reticence is the occurrence of local rumors surrounding “blood stealing” or “blood selling.” Such fears can potentially have dire effects on the success of research projects—for example, high dropout rates that would invalidate the trial's results—and have ethical implications related to cultural sensitivity and informed consent. Though commonly considered as a manifestation of the local population's ignorance, these rumors represent a social diagnosis and a logical attempt to make sense of sickness and health. Born from historical antecedents, they reflect implicit contemporary structural inequalities and the social distance between communities and public health institutions. We aim at illustrating the underlying logic governing patients' fear and argue that the management of these beliefs should become an intrinsic component of clinical research.

Collecting blood samples from individuals recruited into clinical research projects in sub-Saharan Africa can be challenging. Strikingly, one of the reasons for participant reticence is the occurrence of local rumors surrounding “blood stealing” or “blood selling”^1^–^5^; such fears can potentially have dire effects on the success of research projects—for example, high dropout rates that would invalidate the trial's results—and ethical implications related to cultural sensitivity and informed consent. Though commonly considered as a manifestation of the local population's ignorance and/or disconnection from modern society, these rumors often represent an attempt to make sense of sickness and health. As such, the currently adopted solution to address such concerns—namely, including additional information on the medical procedures in the informed consent process—is inadequate to prevent or dispel existing doubts or distrust toward clinical research and fails to circumvent the associated pitfalls for trial implementation and the related ethical hazards. We aim at illustrating the underlying logic governing patients' fear of providing blood samples in Gabon, while showing its applicability in other settings in sub-Saharan Africa by drawing from ethnographic data collected in Gabon between 2007 and 2009 and a general literature review.

The fear of giving blood has been reported across sub-Saharan Africa and has been associated with conceptions of blood as a lifeforce,^4^–^6^ with beliefs that a lack of blood is a sign of diminished strength and inherent disease^7^–^10^ and with notions of blood being a tradable good, requiring remuneration.^4^ In still other settings, such as in Gabon and certain other African countries, these fears are often linked to mystical realms of reality. To understand the logic guiding accusations of blood stealing and selling here we must first understand how and why sudden and unexpected illness/death is frequently attributed to sorcery. In Gabon, disruptions of social order and/or health, such as inexplicable good or bad fortune or sudden illness and death are ascribed to *evou*, a mystical agent of the invisible world, translated in French as *le vampire*, though unrelated to the Western concept of the “living-dead.” The *vampire* is passively available in most people, but is an active force in sorcerers (who use it to inflict illness) and in healers (who with it offer recovery of health).

When sorcery is identified as the cause of an inexplicable event, the question of who is responsible soon follows. In various contexts throughout Africa, Gabon included,^11^–^18^ these accusations are frequently directed at those who are “unnaturally” successful. Such people of influence are suspected of having acquired their power through the consumption of the souls of mystically killed innocents (i.e., “eating people,” “drinking their blood”)^19^–^22^; notably, the agent needed to acquire such unnatural wealth is precisely what clinical trials require from their participants: *blood*. Alleged *evou* users are believed to acquire power through blood sacrifices, consisting of the mystical forfeit of family members, children, and unknown innocents who, as a result, will become ill and die.^19^–^21^,^23^ Consequently, institutions where blood is regularly collected—namely hospitals and research centers—are considered highly suspect.

In the Gabonese context, it is believed that blood drawn from hospital patients or research participants is sold by medical staff to the Rosicrucian Order,^19^–^21^ a semi-secret society affiliated with the Free Masons of Western Europe and the United States. The Rosicrucian order has been strongly linked to colonial^24^ and postcolonial governments,^25^ and boasts numerous highly visible and influential members, reportedly even in government circles. Most importantly, its affiliates are believed to secure their stations through sorcery, whereby members use the acquired blood in sacrificial rites to secure or enhance their positions of influence.^26^

Notably, such perceptions are not a new phenomenon. Rumors of blood theft, including specific references to the medical field, date back to colonial Africa where blood thieves were often described as white people, or their black collaborators, operating at night using European technology, such as medicines and syringes, to extract local people's blood, which they then either sold or transformed into other commodities, such as medicine.^27^–^30^ Among many examples recorded by historians across sub-Saharan Africa, the following are quite illustrative of populations' interpretations of the medical field: in the mid-1940s it was said that Medical Department trucks patrolled the streets in Lamu, Kenya, and, were they to come upon a straggler, would draw out all of his blood with a rubber pump, leaving the body in the gutter.^29^ Over a decade later, “motor vehicles painted red” were said to drain the blood from lone pedestrians captured along the Kisumu highway to Busia; the blood was then reportedly taken to blood banks in hospitals.^29^

Apart from these historical antecedents, various factors continue to foster an association between rumors of blood stealing/selling and medical research in sub-Saharan Africa. Unavoidably, clinical research involves blood sampling from patients. Study participants' poor understanding of the reasons for blood collection and of general trial goals in general increases the ambiguity required for blood taking to be linked to blood theft and inexplicable illness. Furthermore, limited community participation, the social distance between communities and medical institutions, the association of hospitals—and research centers in particular—with authoritarian and elitist state and foreign institutions, and palpable social class divisions further nurture these suspicions. Pre-existing patients' fears within any of these domains may be aggravated by poor comprehension during the informed consent process,^30^ potentially caused, among other factors, by poor communication between the patient and the researcher, power unbalance, lack of time, or by insufficient consideration for the local cultural features in the informed consent process.

Blood stealing/selling, however, is not the only rumor associated with clinical research. Rumors of purposive sterilization of women associated with clinical trials have been reported since 1920 throughout Africa. Furthermore, anti-malarials, vaccinations, condoms, micronutrients, vitamins, and other public health interventions continue to be suspected of causing sterility or of containing hidden contraceptives.^30^–^39^ These rumors continue to be reinterpreted or generated in contemporary African societies and are frequently linked to magical realms of power.^13^–^18^ These beliefs paint a grim historical picture on how local populations perceive medical expertise and therapeutic power, with the underlying concerns likely preceding the advent of clinical research. In this context, health education campaigns or consent forms, meant to inform local communities on the use of collected blood may have little impact as perceptions of blood extraction date back to colonialism, to the introduction of western medicine and persist in modern forms of sorcery accusations. Rumors, such as these, are a reflection of social injustice and asymmetric power relations,^32^ historical threats to the collective survival of particular groups, and a way to interpret society.^31^ As such, though the Gabonese concept of “*evou*” is local, it is founded upon a universal rational search for causality in the face of inexplicable events such as illness and death. Not very different from attributing the inexplicable to the will of God (in monotheist religions), to transgressions or good deeds in a previous life (reincarnation), to the intervention of ancestors (ancestor worship), to spirits (animism), to good luck, faith, or sorcery,^22^,^40^ such universal beliefs represent a social diagnosis or a rational attempt to make sense of a chaotic, unjust and an often incomprehensible world.

Concerns such as these, further present an important bottleneck for the ethical conduct of research and for routine surveillance. The ethical principle of respect for persons, widely recognized as a pillar of medical research,^41^ implies the specific duty of being sensitive to other cultural perspectives.^42^ Researchers should, therefore, develop culturally appropriate ways to communicate with research participants^42^–^44^ and address concerns such as those mentioned previously. Being part of the sociocultural context, similar beliefs are also shared to a large extent by local medical staff within international research teams, making the need for cultural sensitivity all the more pressing. Unfortunately, there is a gap between principles and practices as the adequate expertise and resources needed to address culturally based concerns are still largely absent and remain a non-priority in biomedical and clinical research.

There have been noteworthy—but anecdotal—efforts of researchers trying to counter blood-selling rumors by increasing the information provided to communities or through some form of community participation (e.g., inviting patients to visit the laboratories) and sensitization or even by avoiding the color red in symbols and products related to the intervention. Additional measures such as community consent and participation aiming at reducing the distance between trial teams and communities could have a positive impact. However, no in-depth research on how to avoid or tackle these rumors has been carried out thus far; therefore, making recommendations at this point remains an exercise in futility.

In conclusion, accusations or rumors on blood stealing and blood selling cannot simply be labeled as participants' ignorance. Born from historical antecedents, reflecting implicit contemporary social and structural inequalities, and the social distance between communities and public health institutions, these rumors or accusations represent a social diagnosis and a logical attempt to make sense of the clinical trial in today's world. As such, these beliefs should be acknowledged and their management become an intrinsic component of clinical research.
